# Matrix Metalloproteinases in Relation to Bone Mineral Density: A Two-Sample Mendelian Randomization Study

**DOI:** 10.3389/fgene.2021.754795

**Published:** 2021-11-18

**Authors:** Xin Lv, Pengfei Wu, Shipeng Xiao, Wan Zhang, Yawei Li, Bolin Ren, Zhihong Li, Kun Xia, Bing Wang

**Affiliations:** ^1^ Department of Spine Surgery, The Second Xiangya Hospital, Central South University, Changsha, China; ^2^ Center for Medical Genetics and Hunan Key Laboratory of Medical Genetics, School of Life Sciences, Central South University, Changsha, China; ^3^ Hunan Key Laboratory of Animal Models for Human Diseases, Central South University, Changsha, China; ^4^ Department of Biology, Boston University, Boston, MA, United States; ^5^ Department of Orthopedics, The Second Xiangya Hospital, Central South University, Changsha, China; ^6^ Hunan Key Laboratory of Tumor Models and Individualized Medicine, The Second Xiangya Hospital, Central South University, Changsha, China; ^7^ Hengyang Medical School, University of South China, Hengyang, China

**Keywords:** matrix metalloproteinase, bone mineral density, mendelian randomization, genome-wide association study, summary statistics, causal inference

## Abstract

**Background:** We aimed at investigating causal associations between matrix metalloproteinases (MMPs) and bone mineral density (BMD) by the Mendelian randomization (MR) analysis.

**Methods:** From genome-wide association studies of European ancestry, we selected instrumental variables for MMP-1, MMP-3, MMP-7, MMP-8, MMP-10, and MMP-12. Accordingly, we retrieved summary statistics of three site-specific BMD, namely, forearm, femoral neck, and lumbar spine. We conducted an inverse variance weighted MR as the primary method to compute overall effects from multiple instruments, while additional MR approaches and sensitivity analyses were implemented. Bonferroni-adjusted significance threshold was set at p < 0.05/18 = 0.003.

**Results:** Totally, there was no evidence for causal effects of genetically-predicted levels of MMPs on BMD measurement at three common sites. MR results indicated that there were no causal associations of circulating MMPs with forearm BMD (all p ≥ 0.023) by the inverse variance weighted method. Similarly, there were no causal effects of MMPs on femoral neck BMD (all p ≥ 0.120) and MR results did not support causal relationships between MMPs and lumbar spine BMD (all p ≥ 0.017). Multiple sensitivity analyses suggested the robustness of MR results, which were less likely to be biased by unbalanced pleiotropy or evident heterogeneity.

**Conclusion:** We found no evidence for the causal relationship between MMPs and BMD in the European population.

## Introduction

Bone mineral density (BMD) is a key measurement of bone mass and an essential indicator of osteoporosis, which is prevalent in the aging society. In 1994, the World Health Organization gave the diagnosis standard of osteoporosis as 2.5 SD or more below the young adult average value (Kanis, 1994). The main characteristics of osteoporosis include loss of bone mass, deterioration of the bone microarchitecture, decrement of bone strength and increased risk of fractures, which lead to a systemic skeletal disorder with negative consequences on general health and quality of life in post menopause and in old age (Lane, 2006; Vidal et al., 2019; Capozzi et al., 2020). Fractures due to osteoporosis more likely occur on the hip, vertebral body and distal forearm, therefore, the BMD measurements of forearm (FA), lumbar spine (LS) and femoral neck (FN) are always taken by dual-energy X-ray absorptiometry (DXA) in patients to estimate the general risk of osteoporosis. With the continued ageing of the population worldwide, osteoporotic fractures could present an increasing prevalence and thus lead to higher rates of chronic pain, disability and even death in patients, as well as impose a major economic burden on healthcare systems (Sambrook and Cooper, 2006; Catalano et al., 2017). Current studies have found several risk factors that may decrease BMD (Kenny and Prestwood, 2000; Raisz, 2005; Li and Wang, 2018), but overall, the cause of osteoporosis still remains unclear, which brings difficulty in seeking for effective therapy for this disease.

Matrix metalloproteinases (MMPs) are a family of zinc-dependent neutral endopeptidases capable of degrading extracellular matrix components (Johansson et al., 2000). Previous studies have found that MMPs are expressed in bone tissue as key players in the digestion of bone matrix by osteoblasts, and are involved in bone-destructive lesions (Wahlgren et al., 2001; Azevedo et al., 2018; Fatemi et al., 2020), which indicates that MMPs may play a role in the pathogenesis of osteoporosis. It has been reported that the gene polymorphism of MMP-1 was associated with osteoporosis (Liang et al., 2019), and MMP-3 was negatively related to the osteoblast function markers of serum bone-specific alkaline phosphatase and osteocalcin while positively related to the resorptive function marker of serum cross-linked N- telopeptides of type I collagen (Momohara et al., 2005). Increased levels of MMP-7 and 9 in osteoclasts were reported to be associated with rheumatic osteoporosis (Yang et al., 2013), while MMP-8 participated in the healing process as well as embryonic bone development, and may play an important role in the remodeling of extracellular matrix molecules during bone and cartilage formation (Sasano et al., 2002). MMP-10 was found strongly expressed in osteoclasts and most mononuclear cells within the marrow and produced in an active form with associated degradation (Bord et al., 1998). Meanwhile, recombinant MMP-12 cleaved the putative functional domains of osteopontin and bone sialoprotein, two bone matrix proteins that strongly influence osteoclast activities, such as attachment, spreading and resorption (Hou et al., 2004). These studies strongly suggested the possibility that MMPs are related to osteoporosis. However, due to current randomized controlled trials which were based on either small samples or observational epidemiological studies, whether changes in MMP levels are correlated with BMD remains controversial.

Genome-wide association studies (GWAS) provide a new perspective for understanding genetic determinants that underlie complex disease. The technique of Mendelian randomization (MR), which employs single nucleotide polymorphism (SNPs) as instrumental variables, has been developed to identify causations between a wide range of risk factors and complex diseases. Unlike traditional observational studies, this analytical tool was less susceptible to confounding and reverse causation (Davey Smith and Hemani, 2014). MR has also been widely used these years to explore the causes of osteoporosis (Larsson et al., 2019; Zheng et al., 2019). Given that MMPs were hypothesized to participate in the development of osteoporosis, here we carried out an MR study to identify whether there existed causal associations between MMPs and BMD.

## Materials and Methods

**FIGURE 1:**
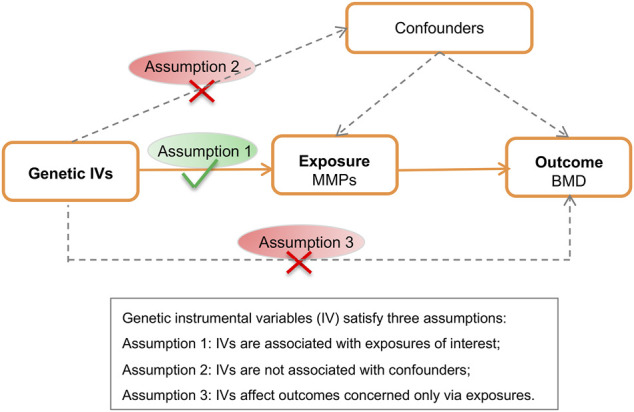
Schematic of the Mendelian randomization analysis. BMD, bone mineral density; MMP; matrix metalloproteinase; SNP, single nucleotide polymorphism.

The MR schematic was shown in Figure 1. There were three underlying assumptions: 1) relevance assumption, genetic instrumental variables are associated with the risk factor of interest; 2) independence assumption, genetic variants are not associated with confounders; and 3) exclusion-restriction assumption, instrumental SNPs influence the outcome concerned only through the risk factor (Burgess et al., 2019). This study utilized publicly accessible datasets from published studies wherein formal consent from participants and ethical approval by committees had been obtained.

### Data Sources

Summary-level association data for MMPs were obtained from GWASs of European ancestry (Salminen et al., 2017; Folkersen et al., 2020). Folkersen et al. (Folkersen et al., 2020) recently conducted a large-scale mapping of protein quantitative trait loci. Circulating levels of MMPs, including MMP-1 (n = 16,889), MMP-3 (n = 20,791), MMP-7 (n = 18,245), MMP-10 (n = 16,933), and MMP-12 (n = 19,178) were measured among a panel of 90 candidate biomarkers related to cardiovascular risk. Summary statistics were released by the SCALLOP consortium (http://www.scallop-consortium.com/scallop_downloads/). Genetic variants associated with MMPs at genome-wide significant significance (*p* = 5 × 10^–8^) and clumped at the threshold (*r* ^*2*^ **= 0.001 within ±1 Mb, EUR 1000 Genomes phase 3) were selected as instrumental variables (Supplementary Table S1). Salminen et al. (Salminen et al., 2017) conducted a GWAS of MMP-8 concentrations in 6,049 Europeans and strongest associations were identified at locus 1q31.3. Two independent SNP associated with MMP-8 meeting the above criteria were utilized as instrumental variables in the ensuing MR analysis. Effect size was given in the unit of SD change in circulating concentration per additional effect allele (Supplementary Table S2–7).

Summary statistics for BMD used in this study were gained from the GWAS datasets released by the GEnetic Factors for OSteoporosis Consortium. Zheng et al. (Zheng et al., 2015) performed a large-scale meta-analysis in 2015 to identify genetic variants associated with BMD including FA-BMD (n = 8,143), FN-BMD (n = 32,735) and LS-BMD (n = 28,498) in individuals of European ancestry from the general population. It is the largest GWAS on DXA-measured BMD so far. The associations for BMD were derived from whole-genome sequencing, whole-exome sequencing, deep imputation, and *de novo* replication genotyping. The association of each SNP with BMD was tested and adjusted for sex, age, square of age and weight. When instrumental SNPs were not present in the BMD datasets, proxies (*r* ^*2*^ > 0.8) were searched and utilized if available. Effect size was given in SDs of BMD in association tests with the additive model (Supplementary Table S2–7). Summary statistics of MMPs and BMD were harmonized in terms of effect allele, and subsequent analyses were based on the merged exposure-outcome dataset.

### Mendelian Randomization

The MR analysis was conducted using the TwoSampleMR (version 0.5.4) package (Hemani et al., 2018) in R 3.6.3 (R Foundation for Statistical Computing, Vienna, Austria). First, individual estimate of the causal effect MMPs on site-specific BMD mediated by each instrumental SNP was computed as the Wald ratio (Walker et al., 2019). Then, the primary method, the inverse variance weighted (IVW) MR was employed to generate overall estimates (Burgess et al., 2013). Two complementary approaches were implemented, considering that IVW estimates would be biased in the presence of invalid instruments or horizontal pleiotropy. Weighted median approach would give robust effect estimates when less than half instruments were invalid (Bowden et al., 2016). MR-Egger regression would serve as a tool to detect unbalanced horizontal pleiotropy, and generate estimates adjusted for pleiotropy (Burgess and Thompson, 2017). IVW estimates were generally more precise, whereas effect estimates given by weighted median and MR-Egger were accompanied by wide confidence intervals (CIs) in the forest plots. Causal effects on BMD were presented in SD units per 1-SD increase in circulating levels of MMPs. The Bonferroni-corrected significance level at *p* < 0.05/18 = 0.003 was adopted in the scenario of multiple tests.

## Results

### Mendelian Randomization Analyses of Matrix Metalloproteinases on FA-Bone Mineral Density

**FIGURE 2:**
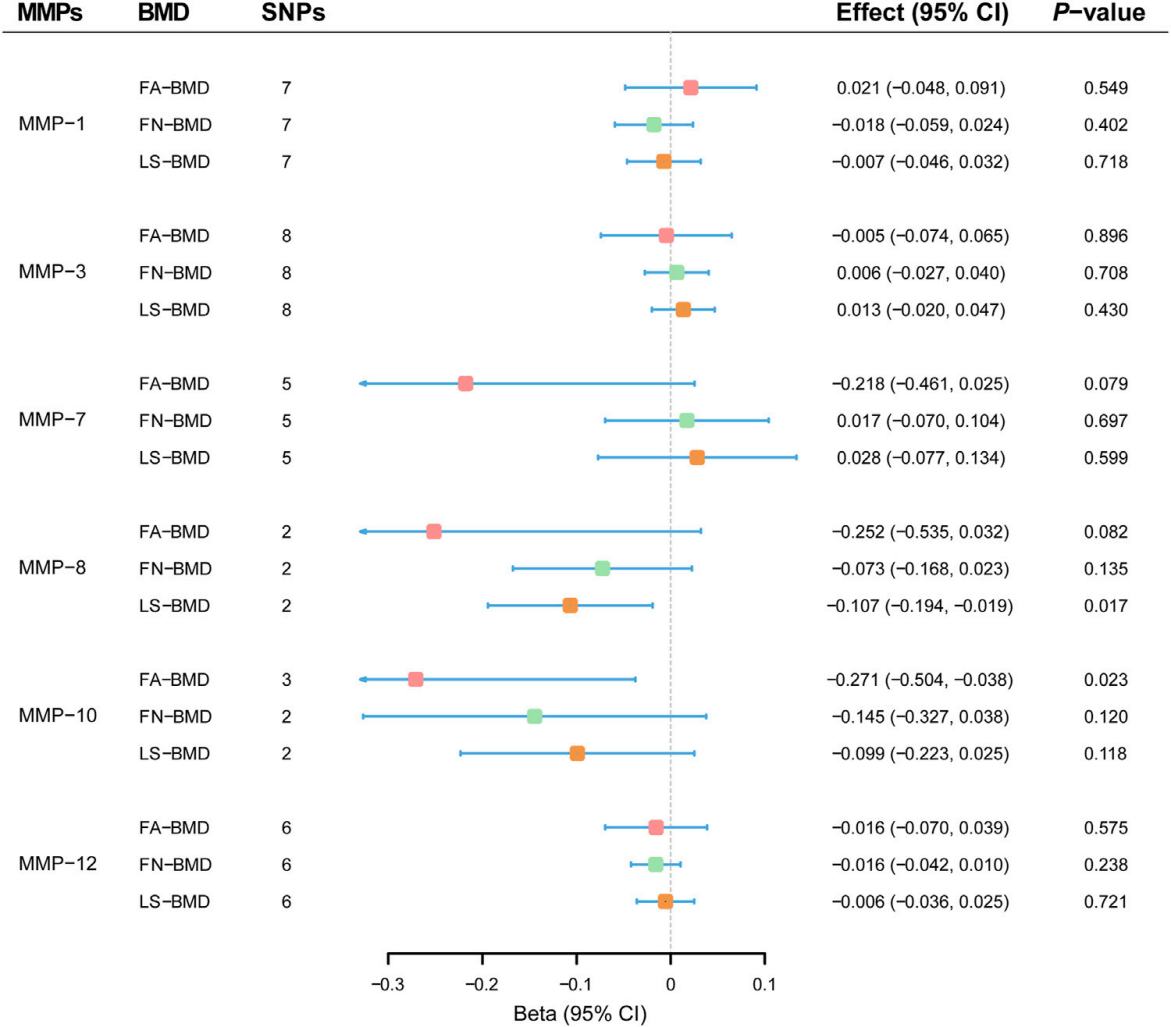
Effect estimates of matrix metalloproteinases on bone mineral density in the Mendelian randomization study. BMD, bone mineral density; CI; confidence interval; FA, forearm; FN, femoral neck; LS, lumbar spine; MMP; matrix metalloproteinase; SNP, single nucleotide polymorphism.

MR results demonstrated that genetically-predicted levels of MMPs were not associated with changes in FA-BMD (Figure 2). By the primary method, causal effects on FA-BMD were 0.024 SD (−0.018–0.402, *p* = 0.402) per 1-SD increase in MMP-1 levels, −0.005 SD (−0.074–0.065; *p* = 0.896) per 1-SD increase in MMP-3 levels, −0.218 SD (−0.461–0.025; *p* = 0.079) per 1-SD increase in MMP-7 levels, −0.252 SD (−0.535–0.032; *p* = 0.082) per 1-SD increase in MMP-8 levels, -0.271 SD (−0.504–−0.038; *p* = 0.023) per 1-SD increase in MMP-10 levels, and −0.016 SD (−0.070–0.039; *p* = 0.575) per 1-SD increase in MMP-10 levels. MR results were generally consistent among causal estimates given by IVW methods and two additional approaches (Supplementary Table S8). In MR analyses with three or more instrumental variables (except for MMP-8), no horizontal pleiotropy was detected according to MR-Egger intercepts and no evident heterogeneity was identified (Supplementary Table S8).

### Mendelian Randomization Analyses of Matrix Metalloproteinases on FN-Bone Mineral Density

Overall, MR estimates suggested that circulating concentrations of MMPs were not associated with FN-BMD. As shown in Figure 2, there was no evidence for causal effects of MMP-1 (−0.018 SD; −0.059–0.024; *p* = 0.402), MMP-3 (0.006 SD; −0.027–0.040; *p* = 0.708), MMP-7 (0.017 SD; −0.070 to 0.104; *p* = 0.697), MMP-8 (−0.073 SD; −0.168–0.023; *p* = 0.135), MMP-10 (−0.145 SD; −0.327–0.038; *p* = 0.120) and MMP-12 (−0.016 SD; −0.042–0.010; *p* = 0.238) by the IVW approach. Complimentary methods further verified the robustness of MR results by the primary method, and there was no evidence for the existence of unbalanced horizontal pleiotropy or heterogeneity (Supplementary Table S9).

### Mendelian Randomization Analyses of Matrix Metalloproteinases on LS-Bone Mineral Density

MR analyses showed that genetically-predicted MMPs were not in relation to LS-BMD (Figure 2). Causal relationships between circulating levels of MMP-1 (−0.007 SD; −0.046–0.032; *p* = 0.718), MMP-3 (0.013 SD; −0.020–0.047; *p* = 0.430), MMP-7 (0.028 SD; −0.077–0.134; *p* = 0.599), MMP-8 (−0.107 SD; −0.194–−0.019; *p* = 0.017), MMP-10 (−0.099 SD; −0.223–0.025; *p* = 0.118) and MMP-12 (−0.006 SD; −0.036–0.025; *p* = 0.721) and measurement in LS-BMD were not significant by the IVW method. According to sensitivity analyses (Supplementary Table S10), MR results by different methods were consistent; besides, unbalanced horizontal pleiotropy or obvious heterogeneity was not present.

## Discussion

Osteoporosis is a common cause of morbidity and mortality worldwide especially in people aged over 60 years. Studies have shown that for decrease per 10 percent in bone mineral density, the risk of fracture increases 2–3 folds (Nguyen et al., 1993), and the mortality rate of patients caused by hip and spine fractures increases to 10–20% (Ioannidis et al., 2009). The causes of decrease in BMD have always been discussed in order to benefit for seeking effective therapy, and more and more risk factors are being identified to better predict the occurrence of osteoporosis and therefore avoid the severe complications of fracture.

The family of matrix metalloproteinases have been considered involved in basic pathological processes of osteoporosis for acting as key roles in the digestion of bone matrix by osteoblasts (Azevedo et al., 2018). However, different studies showed conflict results. For example, Zuo et al. (Zuo et al., 2020) found that MMP-8 was involved in the 17β-Estradiol replacement therapy for postmenopausal osteoporosis, while Viljakainen et al. (Viljakainen et al., 2017) found that there was no significant correlation between MMP-8 levels and low BMD. MR is an effective tool for identifying the causal association between certain exposure and disease while circumventing confounders, which might be the main cause of these inconsistent results. In the recent 3 years, a lot of factors that had been reported related to osteoporosis before have been re-evaluated by MR. Some were further confirmed to be associated with BMD, for instance, serum calcium (Sun et al., 2021), sex hormone-binding globulin (Qu et al., 2021) and age at menarche (Magnus et al., 2020), while others such as vascular endothelial growth factor, uric acid and serum vitamin D got no evidence for their correlations with osteoporosis (Lee and Song, 2019; Sun et al., 2019; Keller-Baruch et al., 2020). In a previous MR study of heel-ultrasound estimated BMD (Folkersen et al., 2020), there were no causal effects of MMP-1, 3, 7, 10, 12 in the European population. In this study, we found no evidence for the causal relationship between MMPs and DXA measured BMD at three common sites.

There are some limitations in present study. First, we could not identify the non-linear relationship between MMPs and BMD. Second, we only evaluated the effect of a small set of MMPs on BMD, but missed such types as MMP-2, -9 and -13, which might be in relation to osteoporosis according to previous studies (Bolton et al., 2009; Zheng et al., 2018). Further MR studies were warranted when relevant datasets are available. Third, it is noteworthy that our study was limited to the effect of circulating MMP levels on BMD, but the intracellular function of MMPs cannot be denied. Forth, both association data of MMPs and BMD were obtained from Europeans in this study. We should be cautious when generalizing the conclusion to other populations.

In this study, we found no evidence for causal relationships between MMPs (MMP-1, 3, 7, 8, 10, 12) and BMD in the European population.

## Data Availability

The original contributions presented in the study are included in the article/Supplementary Material, further inquiries can be directed to the corresponding author.
