# Supplementary material for: Matrix Metalloproteinases in Relation to Bone Mineral Density: A Two-Sample Mendelian Randomization Study
Source: Front Genet. 2021 Nov 18;12:754795. doi: 10.3389/fgene.2021.754795 (PMC8637623; doi:10.3389/fgene.2021.754795)
Supplement: Supplementary file 1 [file DataSheet1.PDF]

# Supplementary Material


**Supplementary Table 1. Data sources of the Mendelian randomization study ........ 2**

**Supplementary Table 2. Summary statistics utilized in the Mendelian randomization study of MMP-1 on BMD........ 3**

**Supplementary Table 3. Summary statistics utilized in the Mendelian randomization study of MMP-3 on BMD........ 5**

**Supplementary Table 4. Summary statistics utilized in the Mendelian randomization study of MMP-7 on BMD........ 7**

**Supplementary Table 5. Summary statistics utilized in the Mendelian randomization study of MMP-8 on BMD........ 9**

**Supplementary Table 6. Summary statistics utilized in the Mendelian randomization study of MMP-10 on BMD........ 10**

**Supplementary Table 7. Summary statistics utilized in the Mendelian randomization study of MMP-12 on BMD........ 11**

**Supplementary Table 8. Mendelian randomization results for causal effects of matrix metalloproteinases on FA-BMD ........ 13**

**Supplementary Table 9. Mendelian randomization results for causal effects of matrix metalloproteinases on FN-BMD ........ 15**

**Supplementary Table 10. Mendelian randomization results for causal effects of matrix metalloproteinases on LS-BMD........ 17**

## Supplementary Table 1. Data sources of the Mendelian randomization study

| Trait | Instrumental SNPs | Sample Size | Ancestry | Access Link |
|---|---|---|---|---|
| MMP-1 | 7 | 16,889 | European | https://www.ebi.ac.uk/gwas/studies/GCST90012033 |
| MMP-3 | 8 | 20,791 | European | https://www.ebi.ac.uk/gwas/studies/GCST90012027 |
| MMP-7 | 5 | 18,245 | European | https://www.ebi.ac.uk/gwas/studies/GCST90012056 |
| MMP-8 | 2 | 6,049 | European | https://www.ebi.ac.uk/gwas/studies/GCST005187 |
| MMP-10 | 3 | 16,933 | European | https://www.ebi.ac.uk/gwas/studies/GCST90012050 |
| MMP-12 | 6 | 19,178 | European | https://www.ebi.ac.uk/gwas/studies/GCST90012070 |
| FA-BMD | – | 8,143 | European | https://www.ebi.ac.uk/gwas/studies/GCST005546 |
| FN-BMD | – | 32,735 | European | https://www.ebi.ac.uk/gwas/studies/GCST005544 |
| LS-BMD | – | 28,498 | European | https://www.ebi.ac.uk/gwas/studies/GCST005545 |

**Abbreviations**: BMD, bone mineral density; FA, forearm; FN, femoral neck; LS, lumbar spine; MMP; matrix metalloproteinase; SNP, single nucleotide polymorphism.

**Supplementary Table 2. Summary statistics utilized in the Mendelian randomization study of MMP-1 on BMD**

| Site-specific BMD | SNP | Position | EA/OA | *F*-statistic | Association with MMP-1 | | | Association with BMD | | |
|---|---|---|---|---|---|---|---|---|---|---|
| | | | | | Beta | Se | *P*-value | Beta | Se | *P*-value |
| FA-BMD | rs12141791 | 1:156419786 | A/G | 49 | 0.083 | 0.013 | $6.40 \times 10^{-10}$ | 0.018 | 0.017 | 0.301 |
| FA-BMD | rs12290253 | 11:102642261 | T/C | 211 | -0.200 | 0.015 | $2.00 \times 10^{-41}$ | 0.005 | 0.019 | 0.812 |
| FA-BMD | rs17297226 | 11:102795639 | A/G | 144 | 0.150 | 0.013 | $5.80 \times 10^{-29}$ | 0.023 | 0.019 | 0.228 |
| FA-BMD | rs186356272 | 11:102190948 | T/G | 141 | 0.460 | 0.064 | $9.90 \times 10^{-13}$ | -0.077 | 0.088 | 0.389 |
| FA-BMD | rs2958130* | 12:57140506 | A/G | 44 | 0.076 | 0.012 | $4.60 \times 10^{-11}$ | 0.002 | 0.016 | 0.907 |
| FA-BMD | rs471994 | 11:102697731 | A/G | 1086 | -0.360 | 0.011 | $3.50 \times 10^{-228}$ | -0.007 | 0.016 | 0.685 |
| FA-BMD | rs6993770 | 8:106581528 | A/T | 96 | 0.120 | 0.012 | $4.60 \times 10^{-23}$ | -0.002 | 0.017 | 0.903 |
| FN-BMD | rs12141791 | 1:156419786 | A/G | 49 | 0.083 | 0.013 | $6.40 \times 10^{-10}$ | 0.011 | 0.008 | 0.201 |
| FN-BMD | rs12290253 | 11:102642261 | T/C | 211 | -0.200 | 0.015 | $2.00 \times 10^{-41}$ | 0.008 | 0.009 | 0.384 |
| FN-BMD | rs17297226 | 11:102795639 | A/G | 144 | 0.150 | 0.013 | $5.80 \times 10^{-29}$ | -0.007 | 0.009 | 0.416 |
| FN-BMD | rs186356272 | 11:102190948 | T/G | 141 | 0.460 | 0.064 | $9.90 \times 10^{-13}$ | -0.062 | 0.042 | 0.152 |
| FN-BMD | rs2958130* | 12:57140506 | A/G | 44 | 0.076 | 0.012 | $4.60 \times 10^{-11}$ | -0.017 | 0.008 | 0.034 |
| FN-BMD | rs471994 | 11:102697731 | A/G | 1086 | -0.360 | 0.011 | $3.50 \times 10^{-228}$ | 0.001 | 0.008 | 0.884 |
| FN-BMD | rs6993770 | 8:106581528 | A/T | 96 | 0.120 | 0.012 | $4.60 \times 10^{-23}$ | 0.002 | 0.008 | 0.788 |
| LS-BMD | rs12141791 | 1:156419786 | A/G | 49 | 0.083 | 0.013 | $6.40 \times 10^{-10}$ | 0.003 | 0.010 | 0.768 |

| | | | | | | | | | | |
|---|---|---|---|---|---|---|---|---|---|---|
| LS-BMD | rs12290253 | 11:102642261 | T/C | 211 | -0.200 | 0.015 | $2.00 \times 10^{-41}$ | -0.009 | 0.011 | 0.415 |
| LS-BMD | rs17297226 | 11:102795639 | A/G | 144 | 0.150 | 0.013 | $5.80 \times 10^{-29}$ | -0.009 | 0.010 | 0.419 |
| LS-BMD | rs186356272 | 11:102190948 | T/G | 141 | 0.460 | 0.064 | $9.90 \times 10^{-13}$ | -0.060 | 0.048 | 0.224 |
| LS-BMD | rs2958130* | 12:57140506 | A/G | 44 | 0.076 | 0.012 | $4.60 \times 10^{-11}$ | 0.004 | 0.009 | 0.674 |
| LS-BMD | rs471994 | 11:102697731 | A/G | 1086 | -0.360 | 0.011 | $3.50 \times 10^{-228}$ | 0.006 | 0.009 | 0.507 |
| LS-BMD | rs6993770 | 8:106581528 | A/T | 96 | 0.120 | 0.012 | $4.60 \times 10^{-23}$ | 0.008 | 0.010 | 0.401 |

**Note**: To measure the strength of instrumental variables of MMP-1, nested functions in the *TwoSampleMR* package were utilized to calculate $F$-statistic and $I^2_{GX}$ (99.5%), where $F < 10$ and $I^2 < 90\%$ indicated that overall MR estimates from multiple variants should be interpreted with caution due to potential bias. * For rs2958130, a proxy SNP (rs7980317, chr12:57158183, $r^2 = 0.92$) in the summary statistics of BMD was utilized.

**Abbreviations**: BMD, bone mineral density; FA, forearm; FN, femoral neck; EA/OA, effect allele/Other allele; LS, lumbar spine; MMP-1; matrix metalloproteinase 1; SE, standard error; SNP, single nucleotide polymorphism.

**Supplementary Table 3. Summary statistics utilized in the Mendelian randomization study of MMP-3 on BMD**

| Site-specific BMD | SNP | Position | EA/ OA | *F*-statistic | Association with MMP-3 | | | Association with BMD | | |
|---|---|---|---|---|---|---|---|---|---|---|
| | | | | | Beta | Se | *P*-value | Beta | Se | *P*-value |
| FA-BMD | rs11225452 | 11:102760774 | A/T | 44 | -0.077 | 0.012 | $2.40 \times 10^{-11}$ | -0.020 | 0.019 | 0.293 |
| FA-BMD | rs11668189 | 19:54754103 | A/C | 1086 | -0.056 | 0.010 | $2.70 \times 10^{-8}$ | -0.010 | 0.016 | 0.527 |
| FA-BMD | rs1291326 | 11:102556321 | T/C | 96 | -0.170 | 0.016 | $6.40 \times 10^{-28}$ | -0.002 | 0.027 | 0.943 |
| FA-BMD | rs145541733 | 11:102677129 | T/C | 45 | -0.390 | 0.054 | $8.80 \times 10^{-13}$ | 0.074 | 0.078 | 0.352 |
| FA-BMD | rs2267373 | 22:38600542 | T/C | 30 | 0.057 | 0.010 | $3.00 \times 10^{-9}$ | 0.035 | 0.017 | 0.037 |
| FA-BMD | rs4614414 | 11:103535890 | T/C | 89 | -0.086 | 0.014 | $2.30 \times 10^{-10}$ | 0.031 | 0.019 | 0.113 |
| FA-BMD | rs632478 | 11:102715681 | T/G | 63 | -0.480 | 0.009 | $5.4 \times 10^{-647}$ | 0.004 | 0.015 | 0.822 |
| FA-BMD | rs74973608 | 11:103244932 | A/G | 33 | -0.120 | 0.017 | $5.50 \times 10^{-12}$ | 0.003 | 0.032 | 0.936 |
| FN-BMD | rs11225452 | 11:102760774 | A/T | 55 | -0.077 | 0.012 | $2.40 \times 10^{-11}$ | -0.001 | 0.009 | 0.934 |
| FN-BMD | rs11668189 | 19:54754103 | A/C | 2705 | -0.056 | 0.010 | $2.70 \times 10^{-8}$ | -0.011 | 0.008 | 0.193 |
| FN-BMD | rs1291326 | 11:102556321 | T/C | 41 | -0.170 | 0.016 | $6.40 \times 10^{-28}$ | -0.005 | 0.013 | 0.732 |
| FN-BMD | rs145541733 | 11:102677129 | T/C | 45 | -0.390 | 0.054 | $8.80 \times 10^{-13}$ | 0.029 | 0.040 | 0.475 |
| FN-BMD | rs2267373 | 22:38600542 | T/C | 30 | 0.057 | 0.010 | $3.00 \times 10^{-9}$ | 0.019 | 0.009 | 0.049 |
| FN-BMD | rs4614414 | 11:103535890 | T/C | 89 | -0.086 | 0.014 | $2.30 \times 10^{-10}$ | 0.017 | 0.010 | 0.079 |
| FN-BMD | rs632478 | 11:102715681 | T/G | 63 | -0.480 | 0.009 | $5.4 \times 10^{-647}$ | -0.003 | 0.008 | 0.713 |

| | | | | | | | | | | |
|---|---|---|---|---|---|---|---|---|---|---|
| FN-BMD | rs74973608 | 11:103244932 | A/G | 33 | -0.120 | 0.017 | $5.50 \times 10^{-12}$ | -0.005 | 0.015 | 0.739 |
| LS-BMD | rs11225452 | 11:102760774 | A/T | 55 | -0.077 | 0.012 | $2.40 \times 10^{-11}$ | -0.006 | 0.011 | 0.606 |
| LS-BMD | rs11668189 | 19:54754103 | A/C | 2705 | -0.056 | 0.010 | $2.70 \times 10^{-8}$ | 0.010 | 0.009 | 0.313 |
| LS-BMD | rs1291326 | 11:102556321 | T/C | 41 | -0.170 | 0.016 | $6.40 \times 10^{-28}$ | -0.008 | 0.016 | 0.612 |
| LS-BMD | rs145541733 | 11:102677129 | T/C | 45 | -0.390 | 0.054 | $8.80 \times 10^{-13}$ | 0.012 | 0.047 | 0.808 |
| LS-BMD | rs2267373 | 22:38600542 | T/C | 30 | 0.057 | 0.010 | $3.00 \times 10^{-9}$ | 0.012 | 0.011 | 0.284 |
| LS-BMD | rs4614414 | 11:103535890 | T/C | 89 | -0.086 | 0.014 | $2.30 \times 10^{-10}$ | 0.006 | 0.011 | 0.565 |
| LS-BMD | rs632478 | 11:102715681 | T/G | 63 | -0.480 | 0.009 | $5.4 \times 10^{-647}$ | -0.006 | 0.009 | 0.475 |
| LS-BMD | rs74973608 | 11:103244932 | A/G | 33 | -0.120 | 0.017 | $5.50 \times 10^{-12}$ | -0.010 | 0.018 | 0.597 |

**Note**: To measure the strength of instrumental variables of MMP-3, nested functions in the *TwoSampleMR* package were utilized to calculate $F$-statistic and $I^2_{GX}$ (99.7%), where $F < 10$ and $I^2 < 90\%$ indicated that overall MR estimates from multiple variants should be interpreted with caution due to potential bias.

**Abbreviation**: BMD, bone mineral density; FA, forearm; FN, femoral neck; EA/OA, effect allele/Other allele; LS, lumbar spine; MMP-3; matrix metalloproteinase 3; SE, standard error; SNP, single nucleotide polymorphism.

**Supplementary Table 4. Summary statistics utilized in the Mendelian randomization study of MMP-7 on BMD**

| Site-specific BMD | SNP | Position | EA/OA | *F*-statistic | Association with MMP-7 | | | Association with BMD | | |
|---|---|---|---|---|---|---|---|---|---|---|
| | | | | | Beta | Se | *P*-value | Beta | Se | *P*-value |
| FA-BMD | rs11607749 | 11:102446374 | T/C | 45 | -0.077 | 0.012 | $4.20 \times 10^{-10}$ | 0.009 | 0.017 | 0.607 |
| FA-BMD | rs17884405 | 11:102398434 | T/C | 308 | -0.220 | 0.018 | $1.90 \times 10^{-37}$ | 0.250 | 0.103 | 0.018 |
| FA-BMD | rs62133135 | 19:54753202 | T/C | 37 | 0.065 | 0.011 | $9.30 \times 10^{-9}$ | 0.011 | 0.016 | 0.510 |
| FA-BMD | rs7946641 | 11:102415001 | A/G | 109 | 0.110 | 0.012 | $5.90 \times 10^{-19}$ | -0.038 | 0.016 | 0.016 |
| FA-BMD | rs9427716 | 1:202012252 | T/C | 69 | 0.087 | 0.012 | $2.00 \times 10^{-12}$ | -0.012 | 0.016 | 0.449 |
| FN-BMD | rs11607749 | 11:102446374 | T/C | 45 | -0.077 | 0.012 | $4.20 \times 10^{-10}$ | -0.006 | 0.008 | 0.488 |
| FN-BMD | rs17884405 | 11:102398434 | T/C | 308 | -0.220 | 0.018 | $1.90 \times 10^{-37}$ | 0.003 | 0.048 | 0.954 |
| FN-BMD | rs62133135 | 19:54753202 | T/C | 37 | 0.065 | 0.011 | $9.30 \times 10^{-9}$ | 0.012 | 0.008 | 0.132 |
| FN-BMD | rs7946641 | 11:102415001 | A/G | 109 | 0.110 | 0.012 | $5.90 \times 10^{-19}$ | -0.002 | 0.008 | 0.755 |
| FN-BMD | rs9427716 | 1:202012252 | T/C | 69 | 0.087 | 0.012 | $2.00 \times 10^{-12}$ | -0.004 | 0.008 | 0.637 |
| LS-BMD | rs11607749 | 11:102446374 | T/C | 45 | -0.077 | 0.012 | $4.20 \times 10^{-10}$ | -0.017 | 0.010 | 0.086 |
| LS-BMD | rs17884405 | 11:102398434 | T/C | 308 | -0.220 | 0.018 | $1.90 \times 10^{-37}$ | -0.046 | 0.053 | 0.399 |
| LS-BMD | rs62133135 | 19:54753202 | T/C | 37 | 0.065 | 0.011 | $9.30 \times 10^{-9}$ | -0.006 | 0.009 | 0.509 |
| LS-BMD | rs7946641 | 11:102415001 | A/G | 109 | 0.110 | 0.012 | $5.90 \times 10^{-19}$ | -0.004 | 0.009 | 0.631 |

| | | | | | | | | | | |
|---|---|---|---|---|---|---|---|---|---|---|
| LS-BMD | rs9427716 | 1:202012252 | T/C | 69 | 0.087 | 0.012 | $2.00 \times 10^{-12}$ | 0.003 | 0.009 | 0.709 |

**Note**: To measure the strength of instrumental variables of MMP-7, nested functions in the *TwoSampleMR* package were utilized to calculate *F*-statistic and $I^2_{GX}$ (99.3%), where $F < 10$ and $I^2 < 90\%$ indicated that overall MR estimates from multiple variants should be interpreted with caution due to potential bias.

**Abbreviation**: BMD, bone mineral density; FA, forearm; FN, femoral neck; EA/OA, effect allele/Other allele; LS, lumbar spine; MMP-7; matrix metalloproteinase 7; SE, standard error; SNP, single nucleotide polymorphism.

**Supplementary Table 5. Summary statistics utilized in the Mendelian randomization study of MMP-8 on BMD**

| Site-specific BMD | SNP | Position | EA/ OA | *F*-statistic | Association with MMP-8 | | | Association with BMD | | |
|---|---|---|---|---|---|---|---|---|---|---|
| | | | | | Beta | Se | *P*-value | Beta | Se | *P*-value |
| FA-BMD | rs1560833 | 1:153334525 | A/G | 59 | -0.155 | 0.020 | $5.31 \times 10^{-15}$ | 0.007 | 0.017 | 0.693 |
| FA-BMD | rs800292* | 1:196642233 | A/G | 151 | -0.241 | 0.020 | $2.42 \times 10^{-35}$ | 0.085 | 0.019 | 0.001 |
| FN-BMD | rs1560833 | 1:153334525 | A/G | 59 | -0.155 | 0.020 | $5.31 \times 10^{-15}$ | 0.001 | 0.008 | 0.990 |
| FN-BMD | rs800292* | 1:196642233 | A/G | 151 | -0.241 | 0.020 | $2.42 \times 10^{-35}$ | 0.025 | 0.009 | 0.005 |
| LS-BMD | rs1560833 | 1:153334525 | A/G | 59 | -0.155 | 0.020 | $5.31 \times 10^{-15}$ | 0.006 | 0.010 | 0.521 |
| LS-BMD | rs800292* | 1:196642233 | A/G | 151 | -0.241 | 0.020 | $2.42 \times 10^{-35}$ | 0.033 | 0.010 | 0.002 |

**Note**: To measure the strength of instrumental variables of MMP-8, nested functions in the *TwoSampleMR* package were utilized to calculate *F*-statistic and $I^2_{GX}$ (89.5%), where $F < 10$ and $I^2 < 90\%$ indicated that overall MR estimates from multiple variants should be interpreted with caution due to potential bias. * For rs800292, a proxy SNP (rs559350, chr1:196642533, $r^2 = 1.0$) in the summary statistics of BMD was utilized.

**Abbreviation**: BMD, bone mineral density; FA, forearm; FN, femoral neck; EA/OA, effect allele/Other allele; LS, lumbar spine; MMP-8; matrix metalloproteinase 8; SE, standard error; SNP, single nucleotide polymorphism.

**Supplementary Table 6. Summary statistics utilized in the Mendelian randomization study of MMP-10 on BMD**

| Site-specific BMD | SNP | Position | EA/ OA | *F*-statistic | Association with MMP-10 | | | Association with BMD | | |
|---|---|---|---|---|---|---|---|---|---|---|
| | | | | | Beta | Se | *P*-value | Beta | Se | *P*-value |
| FA-BMD | rs3129886 | 6:32410576 | T/C | 32 | -0.071 | 0.013 | $4.40 \times 10^{-8}$ | 0.037 | 0.021 | 0.085 |
| FA-BMD | rs55930209 | 11:102608999 | A/C | 84 | -0.260 | 0.032 | $4.10 \times 10^{-16}$ | 0.014 | 0.042 | 0.742 |
| FA-BMD | rs601338* | 19:49206674 | A/G | 122 | 0.120 | 0.011 | $6.30 \times 10^{-28}$ | -0.044 | 0.016 | 0.006 |
| FN-BMD | rs55930209 | 11:102608999 | A/C | 84 | -0.260 | 0.032 | $4.10 \times 10^{-16}$ | 0.064 | 0.020 | 0.002 |
| FN-BMD | rs601338* | 19:49206674 | A/G | 122 | 0.120 | 0.011 | $6.30 \times 10^{-28}$ | -0.007 | 0.009 | 0.432 |
| LS-BMD | rs55930209 | 11:102608999 | A/C | 84 | -0.260 | 0.032 | $4.10 \times 10^{-16}$ | 0.015 | 0.024 | 0.534 |
| LS-BMD | rs601338* | 19:49206674 | A/G | 122 | 0.120 | 0.011 | $6.30 \times 10^{-28}$ | -0.017 | 0.011 | 0.126 |

**Note**: To measure the strength of instrumental variables of MMP-10, nested functions in the *TwoSampleMR* package were utilized to calculate $F$-statistic and $I^2_{GX}$ (99.6%), where $F < 10$ and $I^2 < 90\%$ indicated that overall MR estimates from multiple variants should be interpreted with caution due to potential bias. * For rs601338, a proxy SNP (rs676388, chr19:49211969, $r^2 = 0.85$) in the summary statistics of BMD was utilized.

**Abbreviation**: BMD, bone mineral density; FA, forearm; FN, femoral neck; EA/OA, effect allele/Other allele; LS, lumbar spine; MMP-10; matrix metalloproteinase 10; SE, standard error; SNP, single nucleotide polymorphism.

**Supplementary Table 7. Summary statistics utilized in the Mendelian randomization study of MMP-12 on BMD**

| Site-specific BMD | SNP | Position | EA/ OA | *F*-statistic | Association with MMP-12 | | | Association with BMD | | |
|---|---|---|---|---|---|---|---|---|---|---|
| | | | | | Beta | Se | *P*-value | Beta | Se | *P*-value |
| FA-BMD | rs111850229 | 11:102789864 | T/C | 118 | 0.270 | 0.026 | $4.90 \times 10^{-26}$ | 0.031 | 0.037 | 0.422 |
| FA-BMD | rs117953762 | 13:72450627 | T/C | 47 | -0.250 | 0.046 | $3.40 \times 10^{-8}$ | -0.048 | 0.063 | 0.453 |
| FA-BMD | rs12975366* | 19:54759361 | T/C | 59 | 0.080 | 0.011 | $3.70 \times 10^{-14}$ | -0.008 | 0.016 | 0.626 |
| FA-BMD | rs185686655 | 11:102787261 | T/C | 300 | 0.270 | 0.018 | $9.20 \times 10^{-50}$ | -0.050 | 0.036 | 0.175 |
| FA-BMD | rs72981675 | 11:102721251 | T/C | 3194 | -0.770 | 0.014 | $4.8 \times 10^{-640}$ | 0.016 | 0.023 | 0.506 |
| FA-BMD | rs72987587 | 11:102907759 | T/G | 96 | -0.210 | 0.022 | $8.70 \times 10^{-21}$ | -0.035 | 0.034 | 0.314 |
| FN-BMD | rs111850229 | 11:102789864 | T/C | 118 | 0.270 | 0.026 | $4.90 \times 10^{-26}$ | -0.020 | 0.018 | 0.289 |
| FN-BMD | rs117953762 | 13:72450627 | T/C | 47 | -0.250 | 0.046 | $3.40 \times 10^{-8}$ | 0.000 | 0.031 | 0.989 |
| FN-BMD | rs12975366* | 19:54759361 | T/C | 59 | 0.080 | 0.011 | $3.70 \times 10^{-14}$ | 0.006 | 0.008 | 0.485 |
| FN-BMD | rs185686655 | 11:102787261 | T/C | 300 | 0.270 | 0.018 | $9.20 \times 10^{-50}$ | -0.003 | 0.018 | 0.871 |
| FN-BMD | rs72981675 | 11:102721251 | T/C | 3194 | -0.770 | 0.014 | $4.8 \times 10^{-640}$ | 0.009 | 0.011 | 0.449 |
| FN-BMD | rs72987587 | 11:102907759 | T/G | 96 | -0.210 | 0.022 | $8.70 \times 10^{-21}$ | 0.030 | 0.017 | 0.073 |
| LS-BMD | rs111850229 | 11:102789864 | T/C | 118 | 0.270 | 0.026 | $4.90 \times 10^{-26}$ | 0.006 | 0.021 | 0.796 |
| LS-BMD | rs117953762 | 13:72450627 | T/C | 47 | -0.250 | 0.046 | $3.40 \times 10^{-8}$ | -0.024 | 0.036 | 0.524 |
| LS-BMD | rs12975366* | 19:54759361 | T/C | 59 | 0.080 | 0.011 | $3.70 \times 10^{-14}$ | -0.010 | 0.009 | 0.264 |

| | | | | | | | | | | |
|---|---|---|---|---|---|---|---|---|---|---|
| LS-BMD | rs185686655 | 11:102787261 | T/C | 300 | 0.270 | 0.018 | $9.20 \times 10^{-50}$ | 0.012 | 0.022 | 0.582 |
| LS-BMD | rs72981675 | 11:102721251 | T/C | 3194 | -0.770 | 0.014 | $4.8 \times 10^{-640}$ | 0.005 | 0.013 | 0.700 |
| LS-BMD | rs72987587 | 11:102907759 | T/G | 96 | -0.210 | 0.022 | $8.70 \times 10^{-21}$ | 0.007 | 0.019 | 0.706 |

**Note**: To measure the strength of instrumental variables of MMP-10, nested functions in the *TwoSampleMR* package were utilized to calculate $F$-statistic and $I^2_{GX}$ (99.8%), where $F < 10$ and $I^2 < 90\%$ indicated that overall MR estimates from multiple variants should be interpreted with caution due to potential bias. * For rs12975366, a proxy SNP (rs10405357, chr19:54759666, $r^2 = 0.80$) in the summary statistics of BMD was utilized.

**Abbreviation**: BMD, bone mineral density; FA, forearm; FN, femoral neck; EA/OA, effect allele/Other allele; LS, lumbar spine; MMP-12; matrix metalloproteinase 12; SE, standard error; SNP, single nucleotide polymorphism.

**Supplementary Table 8. Mendelian randomization results for causal effects of matrix metalloproteinases on FA-BMD**

| Traits | No. of SNPs | Effect (95% CI) | *P*-value | Heterogeneity $I^2$ | *P*-value | MR-Egger intercept | *P*-value |
|---|---|---|---|---|---|---|---|
| MMP-1 | | | | | | | |
| Inverse-variance weighted | 7 | 0.021 (-0.048, 0.091) | 0.549 | 0 | 0.771 | | |
| Weighted median | 7 | 0.014 (-0.068, 0.095) | 0.742 | | | | |
| MR-Egger regression | 7 | -0.024 (-0.156, 0.108) | 0.738 | | | 0.011 | 0.466 |
| MMP-3 | | | | | | | |
| Inverse-variance weighted | 8 | -0.005 (-0.074, 0.065) | 0.896 | 27.55% | 0.209 | | |
| Weighted median | 8 | -0.007 (-0.071, 0.057) | 0.828 | | | | |
| MR-Egger regression | 8 | -0.044 (-0.140, 0.053) | 0.410 | | | 0.013 | 0.306 |
| MMP-7 | | | | | | | |
| Inverse-variance weighted | 5 | -0.218 (-0.461, 0.025) | 0.079 | 46.45% | 0.113 | | |
| Weighted median | 5 | -0.180 (-0.407, 0.048) | 0.122 | | | | |
| MR-Egger regression | 5 | -1.247 (-2.045, -0.449) | 0.055 | | | 0.093 | 0.081 |

| | | | | | | | |
|---|---|---|---|---|---|---|---|
| MMP-8 | | | | | | | |
| Inverse-variance weighted | 2 | -0.252 (-0.535, 0.032) | 0.082 | 80.72% | 0.023 | | |
| Weighted median | 2 | NA | NA | | | | |
| MR-Egger regression | 2 | NA | NA | | | NA | NA |
| MMP-10 | | | | | | | |
| Inverse-variance weighted | 3 | -0.271 (-0.504, -0.038) | 0.023 | 35.31% | 0.213 | | |
| Weighted median | 3 | -0.272 (-0.496, -0.049) | 0.017 | | | | |
| MR-Egger regression | 3 | 0.105 (-0.374, 0.583) | 0.743 | | | -0.051 | 0.344 |
| MMP-12 | | | | | | | |
| Inverse-variance weighted | 6 | -0.016 (-0.070, 0.039) | 0.575 | 0 | 0.464 | | |
| Weighted median | 6 | -0.017 (-0.076, 0.041) | 0.558 | | | | |
| MR-Egger regression | 6 | -0.017 (-0.102, 0.068) | 0.720 | | | 0.001 | 0.971 |

**Note:** Weighted median and MR-Egger regression methods were available when there were sufficient instrumental variables ($n \geq 3$).

**Abbreviations**: BMD, bone mineral density; FA, forearm; MMP, matrix metalloproteinase; SNP, single nucleotide polymorphism.

**Supplementary Table 9.** **Mendelian randomization results for causal effects of matrix metalloproteinases on FN-BMD**

| Traits | No. of SNPs | Effect (95% CI) | *P*-value | Heterogeneity $I^2$ | *P*-value | MR-Egger intercept | *P*-value |
|---|---|---|---|---|---|---|---|
| MMP-1 | | | | | | | |
| Inverse-variance weighted | 7 | -0.018 (-0.059, 0.024) | 0.402 | 33.98% | 0.169 | | |
| Weighted median | 7 | -0.012 (-0.050, 0.025) | 0.511 | | | | |
| MR-Egger regression | 7 | -0.004 (-0.089, 0.080) | 0.922 | | | -0.003 | 0.732 |
| MMP-3 | | | | | | | |
| Inverse-variance weighted | 8 | 0.006 (-0.027, 0.040) | 0.708 | 28.30% | 0.202 | | |
| Weighted median | 8 | 0.006 (-0.024, 0.036) | 0.694 | | | | |
| MR-Egger regression | 8 | -0.009 (-0.058, 0.041) | 0.748 | | | 0.005 | 0.440 |
| MMP-7 | | | | | | | |
| Inverse-variance weighted | 5 | 0.017 (-0.070, 0.104) | 0.697 | 0 | 0.549 | | |
| Weighted median | 5 | -0.021 (-0.130, 0.089) | 0.713 | | | | |
| MR-Egger regression | 5 | -0.237 (-0.624, 0.150) | 0.316 | | | 0.023 | 0.278 |

| | | | | | | | |
|---|---|---|---|---|---|---|---|
| MMP-8 | | | | | | | |
| Inverse-variance weighted | 2 | -0.073 (-0.168, 0.023) | 0.135 | 61.17% | 0.109 | | |
| Weighted median | 2 | NA | NA | | | | |
| MR-Egger regression | 2 | NA | NA | | | NA | NA |
| MMP-10 | | | | | | | |
| Inverse-variance weighted | 3 | -0.145 (-0.327, 0.038) | 0.120 | 67.14% | 0.081 | | |
| Weighted median | 2 | NA | NA | | | | |
| MR-Egger regression | 2 | NA | NA | | | NA | NA |
| MMP-12 | | | | | | | |
| Inverse-variance weighted | 6 | -0.016 (-0.042, 0.010) | 0.238 | 0 | 0.511 | | |
| Weighted median | 6 | -0.011 (-0.039, 0.017) | 0.430 | | | | |
| MR-Egger regression | 6 | -0.016 (-0.056, 0.024) | 0.469 | | | 0.001 | 0.982 |

**Note:** Weighted median and MR-Egger regression methods were available when there were sufficient instrumental variables ($n \geq 3$).

**Abbreviations**: BMD, bone mineral density; FN, femoral neck; MMP, matrix metalloproteinase; SNP, single nucleotide polymorphism.

**Supplementary Table 10.** **Mendelian randomization results for causal effects of matrix metalloproteinases on LS-BMD**

| Traits | No. of SNPs | Effect (95% CI) | *P*-value | Heterogeneity $I^2$ | *P*-value | MR-Egger intercept | *P*-value |
|---|---|---|---|---|---|---|---|
| MMP-1 | | | | | | | |
| Inverse-variance weighted | 7 | -0.007 (-0.046, 0.032) | 0.718 | 0 | 0.639 | | |
| Weighted median | 7 | -0.006 (-0.051, 0.039) | 0.792 | | | | |
| MR-Egger regression | 7 | -0.042 (-0.116, 0.032) | 0.319 | | | 0.008 | 0.330 |
| MMP-3 | | | | | | | |
| Inverse-variance weighted | 8 | 0.013 (-0.020, 0.047) | 0.430 | 0 | 0.842 | | |
| Weighted median | 8 | 0.014 (-0.021, 0.050) | 0.437 | | | | |
| MR-Egger regression | 8 | 0.014 (-0.034, 0.062) | 0.584 | | | 0.000 | 0.973 |
| MMP-7 | | | | | | | |
| Inverse-variance weighted | 5 | 0.028 (-0.077, 0.134) | 0.599 | 8.68% | 0.357 | | |
| Weighted median | 5 | 0.000 (-0.124, 0.124) | 1.000 | | | | |
| MR-Egger regression | 5 | 0.038 (-0.496, 0.572) | 0.898 | | | -0.001 | 0.973 |

| | | | | | | | |
|---|---|---|---|---|---|---|---|
| MMP-8 | | | | | | | |
| Inverse-variance weighted | 2 | -0.107 (-0.194, -0.019) | 0.017 | 37.98% | 0.204 | | |
| Weighted median | 2 | NA | NA | | | | |
| MR-Egger regression | 2 | NA | NA | | | NA | NA |
| MMP-10 | | | | | | | |
| Inverse-variance weighted | 3 | -0.099 (-0.223, 0.025) | 0.118 | 0 | 0.521 | | |
| Weighted median | 2 | NA | NA | | | | |
| MR-Egger regression | 2 | NA | NA | | | NA | NA |
| MMP-12 | | | | | | | |
| Inverse-variance weighted | 6 | -0.006 (-0.036, 0.025) | 0.721 | 0 | 0.806 | | |
| Weighted median | 6 | -0.005 (-0.038, 0.027) | 0.746 | | | | |
| MR-Egger regression | 6 | 0.006 (-0.038, 0.051) | 0.800 | | | -0.006 | 0.519 |

**Note:** Weighted median and MR-Egger regression methods were available when there were sufficient instrumental variables ($n \geq 3$).

**Abbreviations**: BMD, bone mineral density; MMP, matrix metalloproteinase; LS, lumbar spine; SNP, single nucleotide polymorphism.
